# A fluidic device for the controlled formation and real-time monitoring of soft membranes self-assembled at liquid interfaces

**DOI:** 10.1038/s41598-018-20998-7

**Published:** 2018-02-13

**Authors:** Arturo Mendoza-Meinhardt, Lorenzo Botto, Alvaro Mata

**Affiliations:** School of Engineering and Materials Science, Queen Mary University of London, London, UK

## Abstract

Membrane materials formed at the interface between two liquids have found applications in a large variety of technologies, from sensors to drug-delivery and catalysis. However, studying the formation of these membranes in real-time presents considerable challenges, owing to the difficulty of prescribing the location and instant of formation of the membrane, the difficulty of observing time-dependent membrane shape and thickness, and the poor reproducibility of results obtained using conventional mixing procedures. Here we report a fluidic device that facilitates characterisation of the time-dependent thickness, morphology and mass transport properties of materials self-assembled at fluid-fluid interfaces. In the proposed device the membrane forms from the controlled coalescence of two liquid menisci in a linear open channel. The linear geometry and controlled mixing of the solutions facilitate real-time visualisation, manipulation and improve reproducibility. Because of its small dimensions, the device can be used in conjunction with standard microscopy methods and reduces the required volumes of potentially expensive reagents. As an example application to tissue engineering, we use the device to characterise interfacial membranes formed by supra-molecular self-assembly of peptide-amphiphiles with either an elastin-like-protein or hyaluronic acid. The device can be adapted to study self-assembling membranes for applications that extend beyond bioengineering.

## Introduction

Interfaces are ubiquitous in Nature and technology, where interfacial properties often mediate the formation and function of bulk materials and organisms. In biology, soft permeable interfaces are critical for the functioning of biological systems as simple as individual entities like viral coats^1^, all the way to complex multi-cellular structures such as the blood-brain barrier^2^. The central role of interfaces in biological systems has motivated substantial work in the development of synthetic analogues for applications such as tissue engineering, drug screening, and drug delivery. One promising approach to create such soft permeable interfaces is to rely on the interfacial self-assembly process that occurs when liquid solutions of two different bio-inspired molecules (e.g. a peptide-amphiphile and a charged protein) are brought in contact^3^. The interaction between different molecules at the interface can lead to spontaneous formation of hydrogels or membranes, whose microstructure, permeability and functional properties can be finely tuned by choosing the appropriate molecular building blocks^4^. While in our group we are mostly interested in self-assembling membranes for tissue engineering^5–7^, self-assembling membranes are of interest for a variety of other applications, including energy storage^8^, optoelectrical devices^9,10^, sensors^11^, polymeric actuators^11^, catalysis^12^, and drug delivery^13,14^.

Investigating and characterising materials formed at liquid-liquid interfaces presents several practical challenges. If the interacting solutions are brought in contact in an uncontrolled manner, for instance by adding a drop of one solution into the other, the thickness, permeability and degree of heterogeneity of the membrane depend sensitively on the experimental protocol. This effect occurs because the convective and diffusive transport of reacting macromolecules to the interface is strongly affected by experimental parameters such as the speed with which the drop impacts the second liquid. The result of the ensuing “turbulent” convection is that different parts of the interface experience different transport histories, thus grow at different rates. Observing the kinetics of formation of the membrane is also a major challenge, as membranes formed by adding one reacting liquid to another can display complex morphologies which are not in general aligned with the line of view of the observer (see e.g.^15–18^). The experimental setups reported in the literature for forming and imaging interfacial self-assembly processes^6,15–20^ present limitations in terms of sample uniformity, hindrances to integration in standard microscopes, and limited to no access to the interacting solutions for direct manipulation. Furthermore, most of these systems require large volumes, which is detrimental for the already cost-intensive development of interfacial self-assembling materials.

Here we propose a simple fluidic device that permits the observation of formation and growth of self-assembling membranes made at the liquid-liquid interface as well as the characterisation of their transport properties. In our device, the membrane forms from the controlled coalescence of two air-liquid interfaces in a straight millifluidics open channel. This configuration limits convection currents and leads to the formation of a vertical membrane, whose initial instant of formation can be controlled by acting on the flow rates of the two solutions. Furthermore, the geometry of the device facilitates manipulation and observation of the membrane. We demonstrate the capabilities of the device by controllably assembling and observing the formation kinetics of two membranes: one formed by the interaction of an elastin-like-polypeptide (ELP) with a peptide amphiphile (PA)^6^, and the other by the interaction of hyaluronic acid (HA) with a PA^19^. Additionally, we show the device may be used to characterise the transport properties of an interfacial membrane by observing the change in concentration of a fluorescent molecule on the two sides of the membrane.

## Experimental Section

### Materials

SYLGARD® 184 Silicone Elastomer poly(dimethylsiloxane) (PDMS) was purchased from Dow Corning Corporation. Marenfield Type B 2 mm outer diameter capillary tubes were purchased from VWR International. Fluorescein isothiocyanate-labelled dextran of 20 kDa molecular weight, 37.0% (w/v) aqueous hydrochloric acid, 28.0–30.0% (w/v) aqueous ammonium hydroxide solution, 1.2 MDa hyaluronic acid, W3500 BioReagent water and Grade I 25% (w/v) glutaraldehyde solution were obtained from Sigma-Aldrich. Petri dishes and thickness no. 0 VWR Micro Cover Glasses were purchased from VWR International. Penta-block ELP was obtained from TP Nanobiotechnology (Valladolid, Spain). Peptide amphiphile molecules (C16V3A3K3 and the fluorescently-labelled peptide amphiphile) were synthesised using standard Fmoc protection chemistry and characterized for quality control as previously described^6^. All used PDMS was prepared in the 10:1 monomer-to-crosslinker ratio established in the literature^21^.

The solutions used for the formation of membranes were prepared by dissolving the reacting reagents in W3500 BioReagent (ultrapure) water. These were the penta-block ELP, the peptide amphiphile C16V3A3K3 (PA), and 1.2 MDa hyaluronic acid (HA). The ELP solutions used were prepared at 1% (w/v) concentration, the PA solutions used were prepared at 1% (w/v) concentration, and the HA solutions used were prepared at 1% (w/v) concentration. All solutions were vortexed to facilitate dissolution. The ELP solid was solubilized by cooling below its transition temperature (of 21 °C) to 5 °C prior to vortexing. After solubilizing, the ELP remains in solution even if taken above its transition temperature. The pH of the PA solution used to form PA-ELP membranes was adjusted to 5.5 by addition of 0.5 µl increments of hydrochloric acid or ammonium hydroxide. The pH of the 20 kDa FITC-dextran solution used to test the transport properties of PA-ELP membranes was adjusted to 7 by addition of 0.5 µl increments of hydrochloric acid or ammonium hydroxide.

### Fabrication of the device

**Figure 1:**
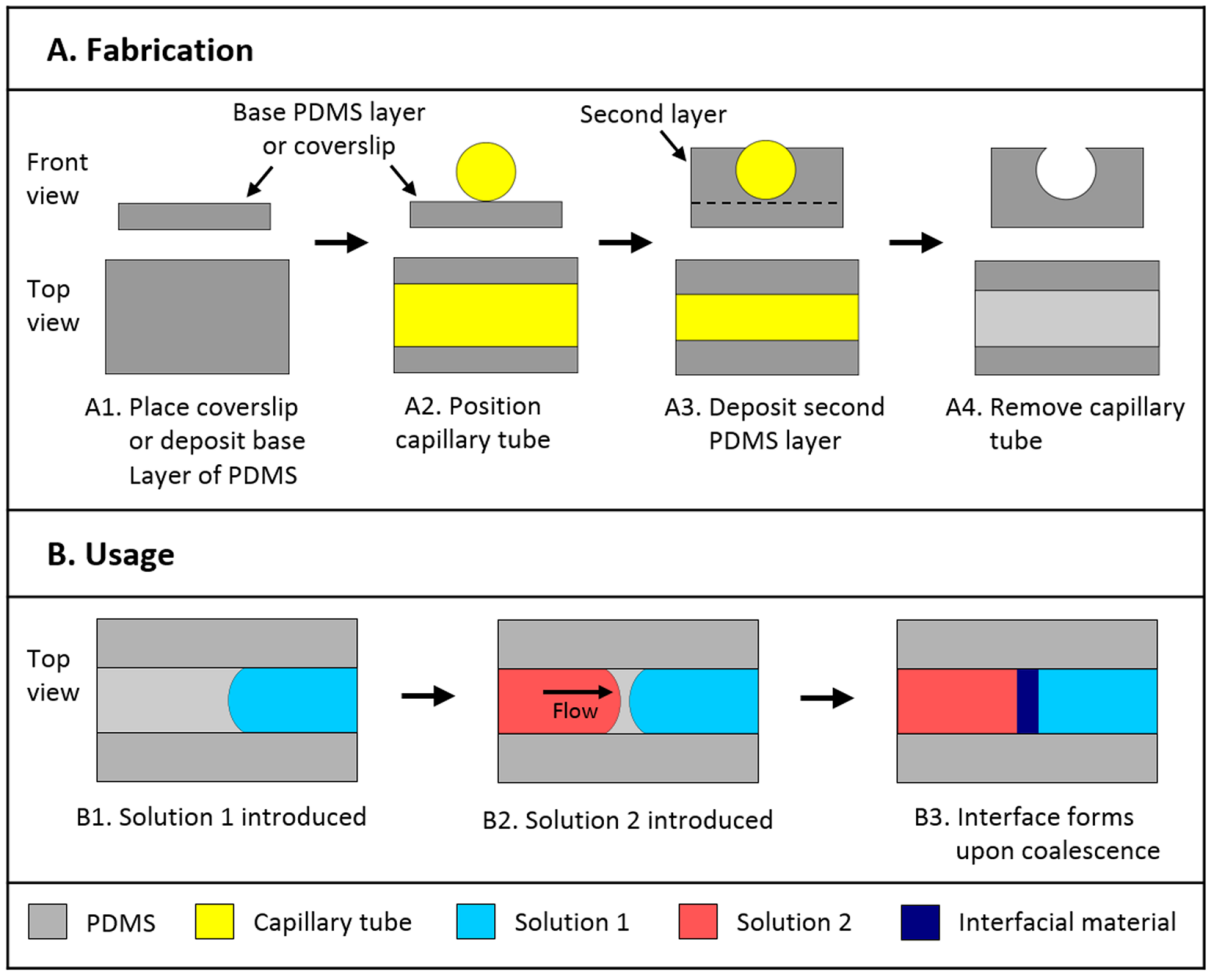
(**A**) Schematic diagrams of fabrication, and (**B**) usage of the device. Channels are fabricated by depositing a base PDMS layer (*A1*). After solidification, a capillary tube is placed on top of the base layer (*A2*). A second PDMS layer is then deposited (*A3*). The capillary tube is removed (*A4*), leaving an open channel. The device is operated by introducing a liquid solution to fill one half of the channel (*B1*). A second solution is introduced from the opposite end (*B2*). Upon coalescence of the two menisci, the solutions react at the liquid interface, forming a membrane via self-assembly of the suspended molecules (*B3*).

The fabrication of the device is presented in Fig. 1, where we illustrate the fabrication of a “thick-floored” PDMS channel. A “thick-floored” channel is robust to manipulation during use. A channel with a thinner base layer (“thin-floored” channel) can also be fabricated. Such a thinner base layer is necessary for optical imaging at high magnifications, where small focal lengths are to be used. A “thin-floored” channel is fabricated similarly to a “thick-floored” channel, except that the second layer is cast directly atop thickness no. 0 VWR micro cover glasses. PDMS was chosen because it is an inert material frequently used in biomedical applications, and its wettability can be easily modified^21^. The device is produced by pouring a first base layer of PDMS (thickness 1 mm) in a Petri dish. After curing at 60 °C for 60 minutes, a glass capillary tube of 2 mm outer diameter is placed on the first layer. A second layer of PDMS is then poured and cured in the dish. The level of PDMS in the second layer must be adjusted to be of the same height as the capillary tube. The Petri dish is then placed in an oven at 60 °C for 24 hours to thoroughly cure both PDMS layers. Removal of the tube with forceps leaves a cylindrical cavity with a rectangular opening at the top. To facilitate the removal of the capillary tube, a scalpel can be used to score the PDMS along the boundary between the capillary and the PDMS. This scoring facilitates the separation of the capillary from the PDMS. Individual channels can be subsequently cut to the desired length.

To facilitate the flow of aqueous solutions along the PDMS channels, the PDMS channels were surface-treated for 60 minutes via UV-Ozone treatment as described by Berdichevsky^22^, using a UVOCS® T10X10/OES (UVOCS Inc.) cleaning system with a mercury lamp emitting light in the 185–254 nm range, and subsequently stored in ultrapure water^21^. All channels were dried with pressurized nitrogen prior to use. This treatment increases the hydrophilicity of the PDMS surface, thereby improving its wettability to aqueous solutions. The curing time of 24 hours used in the preparation of the channels was chosen in order to prevent significant hydrophobic recovery of the PDMS channels following surface treatment^23^.

### Formation of Membranes

Membranes were formed via a two-step process (Fig. 1B): first a drop of the less viscous solution is pipetted into the channel, then a drop of the second solution is pipetted from the opposite end of the channel. The volume of the injected drop depends on the size of the channel. For channels of 2 mm internal diameter, we use drops of 10 µl. The membranes imaged by confocal microscopy (Sec. 3.3) were formed in “thin-floored” channels, while the others were formed in “thick-floored” channels (Sec. 2.2). After initial formation, the membranes used in SEM imaging (Sec. 3.2) and in imaging of FITC-dextran diffusion (Sec. 3.4) were placed in an incubator with controlled 50% humidity at 25 °C, where they were allowed to grow for 24 hours. This procedure increases the mechanical robustness of the membranes. In order to prevent evaporation of the solutions during membrane growth, the membranes were kept inside a Petri dish surrounded by water and sealed with Parafilm M® (Bemis Company, Inc.). All membranes were formed in an incubator with a controlled temperature of 25 °C.

### Bright field Imaging of Membrane Development

Bright field images of the formation of membranes in our device (Sec. 3.2) were obtained using an SP27V-3M Biological Compound Microscope (Brunel Microscopes Ltd) with the recording software Scopephoto (Hangzhou Scopetek Opto-Electric Co. Ltd).

### Confocal Imaging

The 532 laser line of the spinning disk confocal microscope of the PerkinElmer UltraView imaging system (PerkinElmer Inc.) was used to image the cross-section of 5 PA-ELP membranes formed in thin-floored PDMS channels. These membranes were formed using 1% (w/v) PA solution in which fluorescently-labelled peptide amphiphile was also included, at a concentration of 0.01% (w/v). Confocal images of membrane growth were captured 30 seconds, 10 minutes, 1 hour, and 6 hours after coalescence. The thicknesses shown in the captured images were then measured using the image analysis software ImageJ.

### Epifluorescence Imaging of Diffusion Phenomena

Epifluorescence images of the diffusion of FITC-labelled 20 kDa dextran across a PA-ELP membrane were captured using a Leica DMI4000B Epifluorescence Microscope with a LEICA DFC300 FX CCD camera using the FITC filter of the microscope. (PerkinElmer Inc.). After being incubated for 24 hours as described in Sec. 2.3, the membrane was washed four times with ultrapure water to remove unreacted ELP and PA molecules in the solutions surrounding it. The volume of liquid was then adjusted on both sides of the membrane until the distance from the membrane to each of the menisci of the liquid was 10 mm. Subsequently, 10 µl of 1% (w/v) aqueous solution of 20 kDa FITC-labelled dextran was introduced into the channel and made to coalesce with the water on the concave side of the membrane. Epifluorescence images were captured upon coalescence of the dextran solution with the water on the concave side of the membrane, as well as 18 hours, 42 hours, 72 hours, 100 hours, 120 hours, and 144 hours after coalescence. A time-intensity profile was produced by using the image analysis software ImageJ to measure the intensity on the convex side of the membrane in the epifluorescence images.

### Scanning Electron Microscopy

Samples for scanning electron microscopy were prepared by forming membranes using the procedure described in Sec. 2.3. The membranes were fixed in their channels by having their surrounding solutions replaced three times with water via manual pipetting, then two times with 2.5% (w/v) aqueous glutaraldehyde in ultrapure water. The membranes were maintained in the glutaraldehyde solution for 24 hours at room temperature. Following this, the fixed membranes were washed again three times with water, before being dehydrated by having their surrounding water replaced with aqueous ethanol of increasing concentration (50%, 70%, 80%, 90%, 97% and 100%). Following dehydration, the membranes, without removal from our device, underwent critical point drying (K850, Quorum Technologies, UK) and were mounted on SEM stubs using carbon tape, where they were sputter coated with gold (15 nm thickness) after being manually torn to expose their cross-sections. Secondary electron SEM imaging was carried out with an Inspect F50 (FEI Comp, The Netherlands) using an objective aperture of 30 µm, working distance of 5 mm, accelerating voltage of 5 kV, and a spot size of 2.5 nm.

## Results and Discussion

### Device design and usage

The device was fabricated by casting PDMS in a petri dish around a cylindrical glass capillary (Fig. 1). PDMS casting was chosen because it permits fast manufacture while requiring minimal equipment, and because it permits the ability to produce a smooth surface finish on the channel that contains the interacting solutions, as required for optical imaging, where other manufacture methods such as direct machining or 3D printing would not be appropriate. Moreover, the PDMS casting process permits one facile control over the diameter of the channel fabricated, by choosing the size of the capillary tube to be used during casting. Removal of the glass capillary upon solidification of the PDMS layer leaves an open channel that can be accessed from the top. In addition, we found that treating the internal surfaces of the channel to render them more wettable to aqueous solutions (see Sec. 2.2) facilitates the flow of the two liquids along the device. If the channel surfaces are left untreated, the solutions have a tendency to overflow the channel at fast flow rates.

The device is operated by injecting two reacting solutions that form a membrane upon contact from the two ends of the channel (Fig. 1B1 and B2). When the two liquid-air menisci coalesce (Fig. 1B3), the molecules dissolved in the two solutions co-assemble and a membrane forms. The solutions can be injected manually by pipetting, or by the use of a syringe pump connected to capillary tubes that are connected to the ends of the channel. The use of an open channel enables air to escape from the two menisci, preventing the formation of bubbles – a problem otherwise encountered if the solutions are made to flow inside closed tubing. The co-assembly between the two component molecules dissolved in solution starts as soon as the menisci coalesce, leading to the formation of a relatively thick, soft nanostructured membrane (Sec. 3.2). The coalescence is a rather gentle process. While small convective mixing occurs in the neighbourhood of the interface when the two menisci coalesce owing to the release of surface tension energy, the effect can be reduced by increasing the viscosity of the two solutions.

The cylindrical geometry of the channel reduces spurious corner effects (e.g. the accumulation of reactant molecules along the corners of channels of square cross section). Using capillaries of different diameters, channels and hence membranes of different sizes can be obtained. Using channels with small diameters could be useful to reduce the amount of interacting solutions. The straight channel shape enables visual and physical access to both sides of the interface, facilitating real-time imaging, analysis, and manipulation.

Compared to previously reported experimental setups^6,15–18,20^ for the formation and imaging of interfacial self-assembling membranes, our device enables the production of membranes exhibiting a controllable, reproducible geometry. The lateral size of the membranes that can be fabricated is set by the channel diameter $D$. The membrane thickness depends on the mass flow rates of reactants towards the interface and how the molecular interactions change as the thickness grows. Therefore, the kinetics of growth depends on the specific reactants used. The diameter $D$ should be small so that the menisci do not collapse under their own weight (a condition of sufficiently small Bond number) but, in principle, there are no lower bounds for $D$. Provided well-resolved membrane images can be taken, it may be possible to work with microscopic fluidic systems as well.

The volumes of the interacting solutions can be controlled, and the contents of the solutions modified (potentially in real-time, thanks to the open channel geometry). In contrast to a device reported by Abhyankar *et al*.^24^, which required the tested self-assembling membrane to be formed in a separate setup, then be placed manually between fluidic channels and then sealed, in our device the membrane is formed *in situ*. In addition to the advantages described above, this feature allows the user to examine small membranes whose manual handling would be challenging. Furthermore, a significant benefit of our setup is that it enables real time observation of the dynamic formation of the membrane.

To demonstrate potential uses of the device to characterise self-assembling membranes, we used two types of interfacial co-assembly systems: a peptide amphiphile/elastin-like-polypeptide (PA-ELP) system^6^ and a peptide amphiphile/hyaluronic acid (PA-HA) system^19^. Both of these systems form soft membranes by self-assembly when aqueous solutions of the two molecules (e.g. ELP and PA) come in contact with each other. We have visualized the formation of the membranes by combining the device with three different imaging configurations: standard bright-field optical microscopy, fluorescence confocal microscopy and epifluorescence microscopy. Standard bright-field optical microscopy and fluorescence confocal microscopy were used to investigate the formation and growth of the test membranes, providing information for qualitative (optical) and quantitative (confocal) analysis. It is important to mention that the device allows for variations in the solutions such as types of molecules and their concentration. In order to demonstrate the reliability and reproducibility of the device, we have used two self-assembling systems that were previously reported. For both PA-ELP^6^ and PA-HA^15,19^ systems, we used 1% (w/v) of the molecules as this concentration has previously been reported to generate optimum membranes. In this way, our study not only validates that the device can be used with different interfacial systems in a reliable manner but also illustrates how it may be used to conduct morphological and kinetic comparisons between the interfacial systems.

### Observation of membrane formation

**Figure 2:**
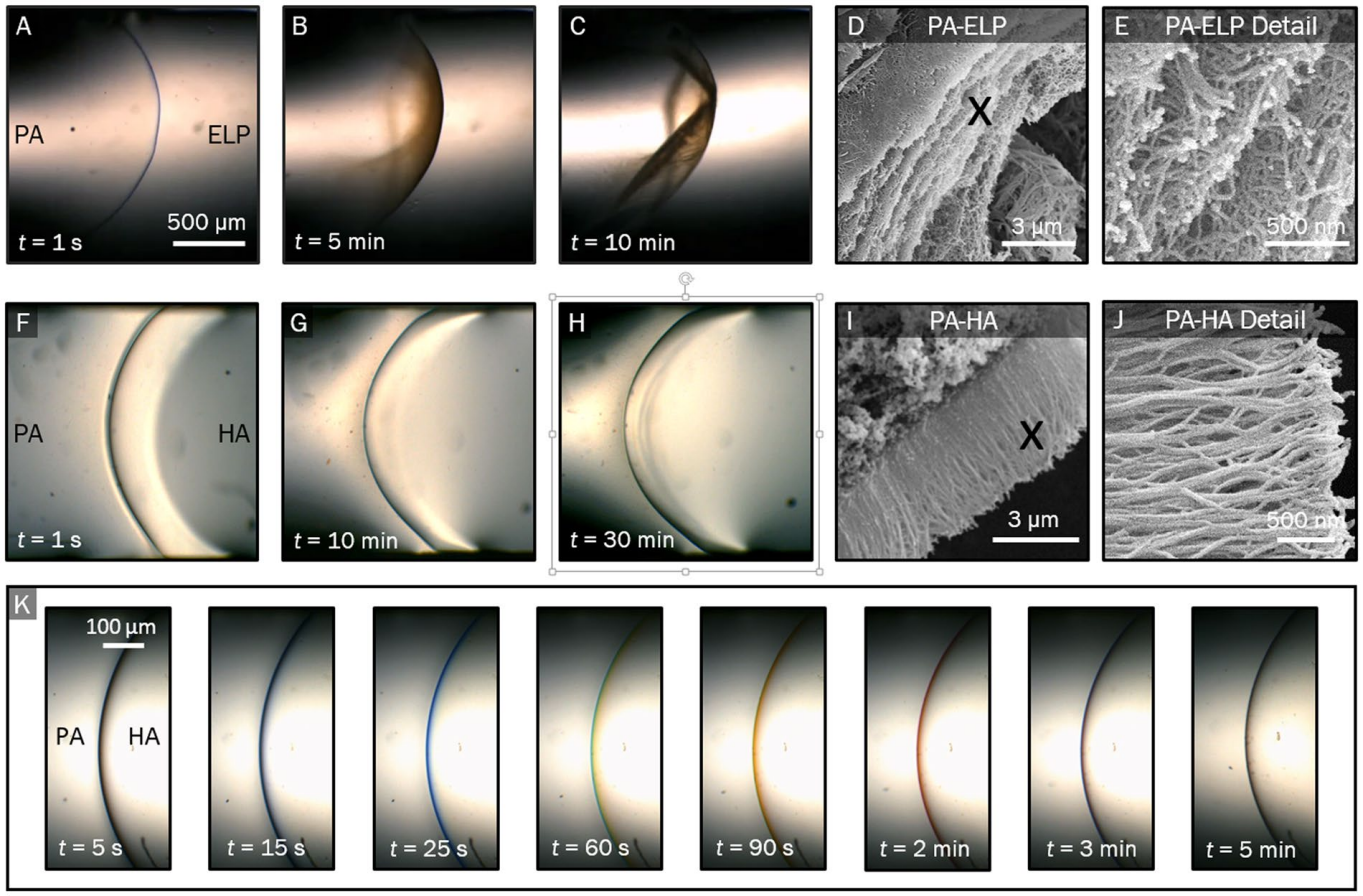
Bright field optical microscopy of (**A–C**) an PA-ELP membrane and (**F–H**) of a PA-HA membrane, illustrating the membrane growth process. Time t = 0 corresponds to the onset of membrane formation. Scanning electron microscopy of cross section and detail of nanofibrous structure of PA-ELP (**D,E**) and PA-HA (**H,J**) membrane, showing multi-layer parallel nanofibrous microstructure and three-layer perpendicular nanofibrous microstructure, respectively. (**I**) Bright field images of first 5 minutes of PA-HA membrane formation, showing a progressive change in colour.

The device enables the real-time monitoring of the formation of interfacial membranes. This feature is illustrated in Fig. 2, where we characterise the time-dependent growth for both PA-ELP and PA-HA membranes. From representative bright field optical microscopy images, it is evident that the PA-ELP system generates a membrane with a curve opposite to the membrane generated by the PA-HA system. In the case of the PA-ELP system, the membrane was observed to always be generated with its concave side facing the PA solution, regardless of the order in which the two solutions were introduced into the device. We believe this is an effect caused by the difference in surface tensions between the two solutions^25^. However, in the case of the PA-HA system, the membrane is generated with its convex side facing the PA solution, which we believe is caused by the large viscosity of the HA solution dominating surface-tension effects.

Both co-assembly systems were observed to grow in our device forming nanofibrillar hierarchical structures (Fig. 2D,E,I,J) similar to those reported in the literature^6,19^. In particular, SEM images (Fig. 2D,E,I,J) indicate a distinctive multi-layered architecture of parallel fibres for the PA-ELP membrane (Fig. 2D,E) as well as a three-region architecture of amorphous HA, fibres parallel to the interface, and fibres perpendicular to the interface for the PA-HA membrane (Fig. 2I,J).

By positioning the meniscus of the first solution in the field of view of the microscope, we were able to accurately control the position at which the interface was formed. This feature allowed us to image the first few seconds of the formation process. The formed membrane adhered tightly to the walls. Attachment of self-assembling membranes to solid boundaries has been previously observed^6,15,17,20^. Interestingly, the membrane is adhesive only during the initial stage of formation. Once the membrane is fully formed, it ceases to be adhesive and can be easily detached and readily manipulated.

The PA-ELP membrane was observed to become increasingly opaque and change colour over time, from blue to brown (Fig. 2A,B,C). In contrast, the PA-HA membrane remained translucent and changed colour only at its edge (Fig. 2K), from blue to black. Interestingly, the membrane changed colour over time from blue to green, yellow, orange, and red, before turning brown and finally black (Fig. 2I). We hypothesize that this variation in colour is caused by optical interference^26^. The light reflected by a thin membrane at small angles to the observer is of a discrete wavelength and colour, determined by the thickness of the membrane. The slower growth rate of the PA-HA membrane hence explains the longer period of time taken by the PA-HA membrane to display a change in colour from blue to brown (5 min) compared to the PA-ELP membrane (1 s). It may hence be possible that imaging during the first second of formation of a PA-ELP membrane would produce the same spectrum of colours as that produced by the PA-HA membrane. Such an experiment would serve to probe whether it is the direction of fibre alignment, other nanostructural features, or solely the membrane thickness, that brings about a progressive change in colour. Imaging in this time scale (less than one second) would require the usage of a high-speed camera.

### Quantification of membrane formation kinetics

**Figure 3:**
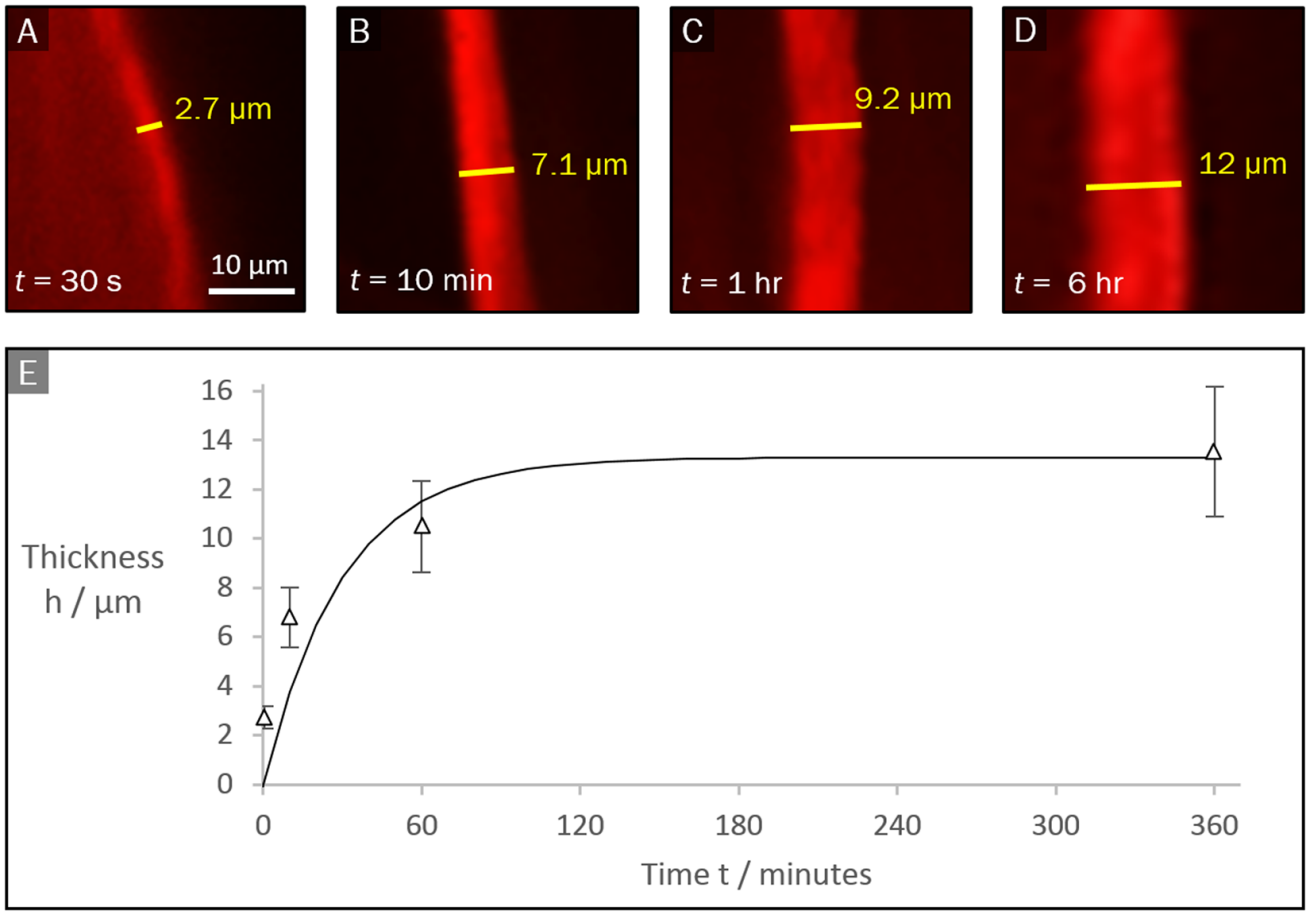
(**A–D**)Fluorescence confocal microscopy images of cross section of PA-ELP membrane visualized by the addition of fluorescently-labelled PA molecules to the PA solution. (**E**) Membrane thickness vs. time. The symbols indicate experimental data. The continuous line is equation (1).

The possibility of tracking the growth of the membrane in real time, without the need to remove the membrane to place it e.g. in an SEM chamber, represents a significant advantage of the current device (particularly when handling soft, fragile, or growing, fast-forming membranes). Figure 3 shows the time evolution of a PA-ELP membrane thickness, as measured by confocal microscopy. The horizontal cross-section was imaged at the apex of the membranes’ curvature in order to reduce inaccuracy in the apparent thickness. The thickness grows smoothly, apparently reaching a plateau for long times. An exponential fit to a function

1 $$h(t)={h}_{\infty }(1-{e}^{-\frac{t}{{\tau }_{k}}})$$

gives a kinetic time scale of formation ${\tau }_{k}\cong 30\,min\,$s and a saturation membrane thickness of ${h}_{\infty }\cong 13.3\,\mu m$. In the conceptual model proposed by Inostroza et al^6^. and Zha *et al*.^27^, the rate of growth of the PA-ELP membrane was related to the time taken for PA molecules to diffuse across the interface, reaching the reactive region where the molecules self-assemble. As the membrane thickness increases, the flux of PA molecules to the reactive region decreases, leading to a finite saturation thickness.

Inostroza *et al*.^6^ reported a saturation thickness of about 30 μm, as measured by SEM after 48 hours, by adding a 10 µl drop of 1% (w/v) PA into a 90 µl drop of 1% (w/v) ELP. The difference in thickness with our result (13.3 μm` measured at 6 hours) is likely due to the time-dependent growth of the membranes, or may result from the different ratios of reactant volumes used in the experiments. Nonetheless, the distinctive hierarchical multilayered structure is similar between the two studies, and the order of magnitude of the obtained thickness is comparable. This suggests that the use of the device does not significantly alter the membrane formation process.

We have developed a simple membrane growth model based on first-order kinetics for the case in which the concentration of the reactants across the membrane is constant (see Supporting Information). This model gives an exponential approach, as in equation(1), provided that the rate constant decreases linearly with the thickness $h$. According to the model the kinetic scale ${\tau }_{k}$ is a function of the mass density of the membrane, the surface concentration of reactants and a coefficient $\beta$ measuring the decrease in the efficiency of the interfacial reaction as the membrane grows. Similar or more complex models can be applied to extract kinetic parameters of membrane formation by fitting to data such as the one presented in Fig. 3E.

### Quantification of diffusion across the membrane

The linear geometry of the device facilitates the characterisation of the transport properties of the membrane, such as effective membrane diffusion or permeability coefficients. Such measurements are more complex to model when using closed membranes such as microcapsule, owing to the finite amount of diffusant present within the membrane and to the complex geometry of the boundary^28^.

**Figure 4:**
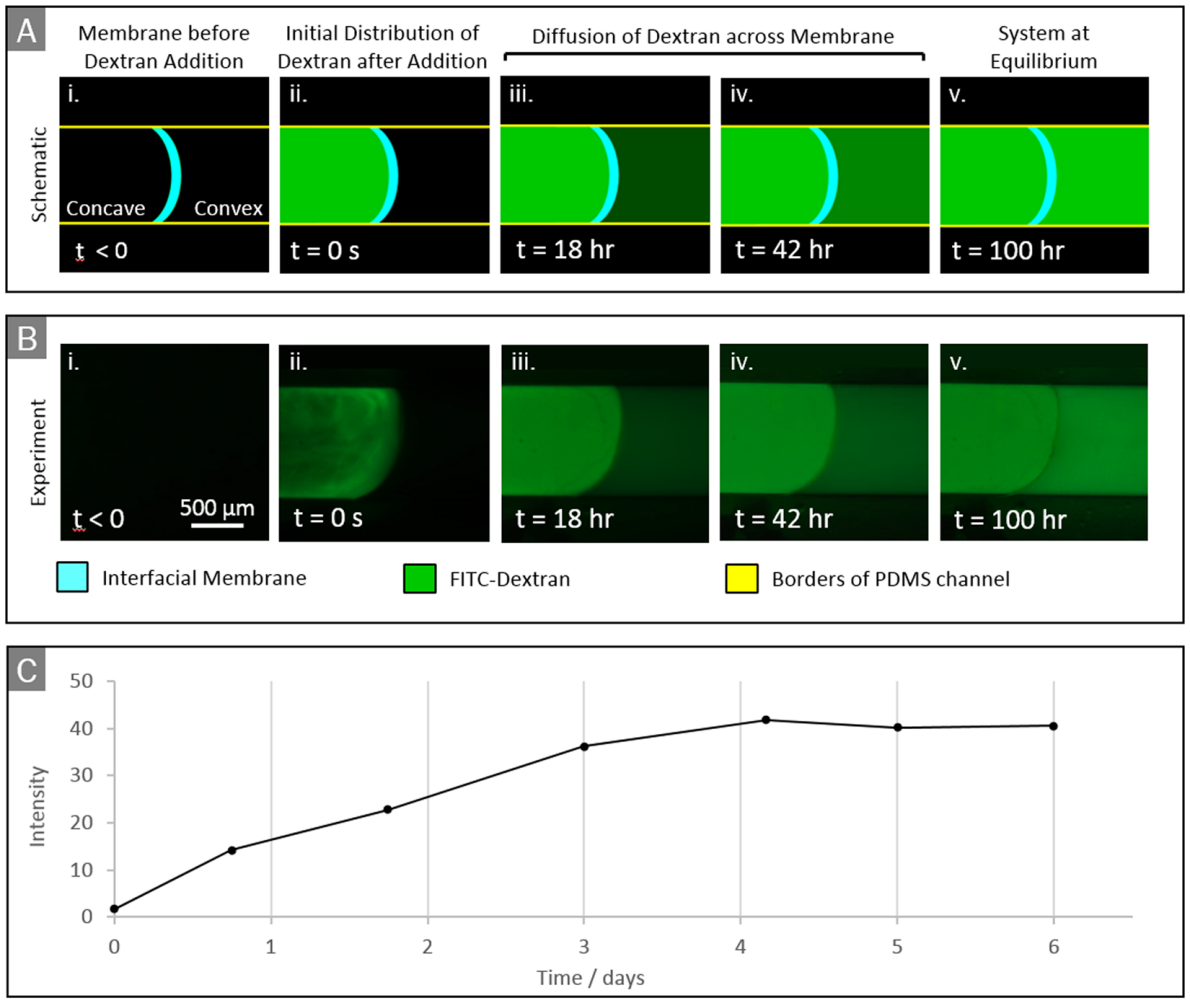
(**A**) Diagram of diffusion experiment using epifluorescence and (**B**) experimental images. The membrane here is PA-ELP. Description: (*i*) ELP and PA solutions are replaced with ultrapure water and membrane is set up for imaging; (*ii*) FITC-labelled Dextran is added to the water on the concave side; (*iii-iv*) Dextran molecules diffuse across the membrane, increasing the concentration on the convex side; (*v*) approximate equilibrium is reached. (**C**) Average fluorescence light intensity on convex side vs. time.

To illustrate the use of our device to characterise the diffusivity of a membrane, we measure the diffusion of FITC-labelled dextran molecules across a PA-ELP membrane (Fig. 4A,B). After replacing the ELP and PA solutions with ultrapure water, the dextran molecules were added to the water on the concave side of the membrane. The dextran molecules rapidly became uniformly distributed within the concave region (Fig. 4B.ii,A.ii) and subsequently diffused across the membrane, increasing the concentration on the convex side. Approximate equilibrium between the concentrations on the two sides was reached in about 100 hours.

We assume that the dextran molecules are much smaller than the membrane pores (average diameter of a dextran molecule ≈13 nm, as provided by the supplier; average pore within the PA-ELP membrane ≈200 µm, as determined from SEM images), so direct sieving by the membrane is not expected. Is important to keep in mind that the measurement of the pore size of the membrane is approximate as hydrated samples are expected to change their structure when prepared for SEM imaging. Nonetheless, we assume that the relatively smaller dextran molecules do not get trapped within the hydrated membrane pores nor disrupt the membrane integrity as a result of charge screening since the pH of the dextran solution was adjusted to 7, which is near its isoelectric point. In addition, we assume that the charges in the interacting molecules had previously been screened for the formation of nanofibres to take place, hence stabilizing them against further electrostatic interactions. Furthermore, no evident leakage regions were observed (Fig. 4B), suggesting tight adherence of the membrane to the channel walls. The time evolution of the fluorescence intensity on the convex side of the membrane is shown in Fig. 4C. In this experiment, the intensity profile was obtained by capturing images at selected times via time-lapse fluorescence microscopy (Fig. 4B) and measuring the average intensity on the convex side of the membrane using the software ImageJ. In agreement with Fig. 4B, the intensity is seen to reach a plateau in about 100 hours.

Assuming that the light intensity is proportional to the dextran concentration, the data in Fig. 4C can be used to extract an effective diffusion coefficient $D$ associated to transport within the membrane. Mass balance for the concentration of dextran molecules on the convex side requires

2 $$\frac{d(V{C}_{cvx})}{dt}=FA$$

where $F$ is the mass flux (per unit area) of dextran molecules across the membrane, $A$ is the area of the membrane, $V$ is the volume of the channel on the convex side and ${C}_{cvx}$ is the corresponding mass concentration of dextran. Assuming one dimensional diffusion, Fick’s law requires $F=-D\frac{\partial C}{\partial x}$, where the derivative is evaluated at the surface of the membrane on the convex side. The concentration gradient is due to a concentration difference ${C}_{cnc}-{C}_{cvx}$ occurring within a thin region of thickness $h$. Therefore, assuming quasi-steady transport within the membrane, $F\cong D({C}_{cnc}-{C}_{cvx})/h$^29^. Here $h$ is the membrane thickness and ${C}_{cnc}$ is the concentration on the concave side. Taking into account that $\frac{d{C}_{cvx}}{dt}\cong -\frac{d{C}_{cvc}}{dt}$ because the convex and concave side of the channel have practically equal volume, equation(1) yields

3 $$\frac{d{C}_{cvx}}{dt}=\frac{{C}_{\infty }-{C}_{cvx}}{{\tau }_{D}},$$

where ${C}_{\infty }\,$is the equilibrium concentration and ${\tau }_{D}=hV/(2DA)$. The solution of^3^ gives an exponential approach to ${C}_{\infty }$ on a time scale ${\tau }_{D}$. In our case ${\tau }_{D}\,$is roughly 100 h, V = 31.4 mm^3^ and A = 3.14 mm^2^, and h = 13.3 µm. As a consequence, the diffusion coefficient for dextran across the specific membrane considered in Fig. 4 can be estimated to be $D=\frac{hV}{2A{\tau }_{D}}\cong 0.18\,\mu {m}^{2}/s$. Indeed, more sophisticated models can be applied. However, the main point here is that the diffusion process in our device is essentially one dimensional, therefore one can apply the rich set of models developed for one-dimensional diffusion processes to characterise the membrane. For example, models for multi-layers are available^30^ that could be adapted to accurately characterise multi-layer membranes.

The solutions surrounding the membrane were replaced with ultrapure water after the membrane was completely formed without causing the membrane to detach. The possibility of replacing the solution adjacent to the interface and manipulating the membranes after its formation (for example with small forceps) is a practically important advantage of our device that could be exploited to characterise a variety of other interfacial self-assembly systems^6,14,15,17–20,31^.

## Conclusion

We have developed a simple device that facilitates the formation, imaging, and manipulation of soft self-assembling membranes. The device is fabricated by casting of PDMS to form a cylindrical open channel, followed by surface treatment. Experiments with two macromolecular self-assembly systems of interest to bioengineering applications were carried out to demonstrate the capabilities of the device. The device enables the formation of membranes that have microstructures similar to those reported in the literature, suggesting that the device does not introduce essential artefacts. To illustrate the capabilities of the device, we characterise the formation kinetics and transport properties of a PA-ELP membrane by combining the device with a standard optical, epifluorescence, and confocal microscopy methods.

The device enables one to control the instant of formation of the membrane, produces membranes that are roughly aligned with the line of view of the observer, and requires very small amounts of reactants. Because in the device the membrane forms upon coalescence of two slowly moving liquid streams, convective effects which can affect the quality of the membrane are limited. The device has other practical advantages, which are discussed in Sec. 3.1.

The device we propose is expected to have general applicability. It could be used to investigate in real time the formation and properties of a variety of interfacial materials, from nanoparticle monolayers^9,10,32^ to lipid bilayers^33^, polyelectrolyte multilayer films^13^, and polymeric films^8,11^, for applications in optoelectrical devices, drug delivery, catalysis, sensors, separation membranes, polymeric actuators, and energy storage and production^34^. Finally, the device could be used to study a variety of molecular co-assembling systems beyond PA-ELP and PA-HA. In addition, it may be possible to introduce a third macromolecule^15^, use a different self-assembling peptide^18^, or use a different macromolecule in place of ELP or HA^20^. A prerequisite is that the reaction occurs at or near the liquid-liquid interface.

### Data Availability

All data generated or analysed during this study are included in this published article (and its Supplementary Information files).

## Electronic supplementary material

Supplementary Information
