# Supplementary material for: A fluidic device for the controlled formation and real-time monitoring of soft membranes self-assembled at liquid interfaces
Source: Sci Rep. 2018 Feb 13;8:2900. doi: 10.1038/s41598-018-20998-7 (PMC5811436; doi:10.1038/s41598-018-20998-7)
Supplement: Supplementary file 1 — Supplementary Information [file 41598_2018_20998_MOESM1_ESM.pdf]

Supporting Information

A fluidic device for the controlled formation and real-time monitoring of soft membranes self-assembled at liquid interfaces

Arturo Mendoza-Meinhardt[a,b] Lorenzo Botto[a,c] Alvaro Mata[a,d]*

a. School of Engineering and Materials Science, Queen Mary University of London
b. +44 (0) 207 882 7303, a.mendozameinhardt@qmul.ac.uk
c. +44 (0) 207 882 3757, l.botto@qmul.ac.uk
*d. +44 (0) 207 882 6279, a.mata@qmul.ac.uk **

Corresponding Author

* To whom correspondence should be addressed

E-mail: a.mata@qmul.ac.uk

Phone: +44 (0)207 882 6279

Table of Contents **Page number**

1. Derivation of model for membrane growth S1

1. Derivation of model for membrane growth

Here we present a simple mathematical model of membrane growth based on first-order kinetics.

Consider two solutions A and B which at time t=0 meet at the interface located at x=0. The solution A is initially located in the region $x < 0$ and the solution B in the region $x > 0$. Denote by $c_A(x,t)$ and $c_B(x,t)$ the bulk concentration of solutes A and B, the problem is predicting the evolution of the membrane thickness $h(t)$ with time.

Mass conservation imposes that the rate of change of the membrane's mass is equal to the mass of the reactants A and B that self-assemble at $x = 0$. Calling ρ the average mass density of the membrane, and S the channel cross section, we can write

$$\rho S \frac{dh}{dt} = r$$

where the reaction rate r is the total mass of reaction products C created by the self-assembly process per unit time at the interface according to the reaction

$$A + B \rightarrow C$$

For a first-order reaction occurring at the interface, we can write

$$r = kS\, c_A(x = 0^-)^{1/2} c_B(x = 0^+)^{1/2}$$

where k is a rate constant having the units of a length per unit time. The interfacial concentrations of A and B in the expression above should be interpreted as the bulk solute concentrations immediately to the left and to the right of the interface. For well mixed conditions we can assume that the interfacial concentrations are equal to the corresponding bulk concentrations.

The rate constant must depend on the bulk solute diffusivities D_A and D_B. For example, if both diffusivities where equal to zero, in the absence of convection the solute molecules would not be able to reach the interface, and the self-assembly process would not take place. It must also somewhat depend on h itself, because the experimental data shows that the growth slows down as the membrane thickness increases.

The simplest model for k is the linear model

$$k = \alpha - \beta h$$

where both the parameters α and β depend on the bulk diffusivities. The parameter α is a measures of how fast the interfacial reaction takes place in the limit $t \rightarrow 0$ in which the membrane is infinitesimally thin. The parameter β is a measures of the decrease in the efficiency of the interfacial reaction as the membrane grows.

The equation governing the membrane growth is thus

$$\rho \frac{dh}{dt} = (\alpha - \beta h) \sqrt{c_{A,i}} \sqrt{c_{A,i}}$$

The solution of this linear equation is $h(t) = \frac{\alpha}{\beta}\left[1 - \exp(-t\beta\sqrt{c_{A,i}c_{B,i}} / \rho) \quad \right]$.

The two parameters of this exponential function above can in principle be obtained experimentally. The ratio $\frac{\alpha}{\beta}$ can be obtained by measuring the final thickness of the membrane h_∞, since $h_\infty = \frac{\alpha}{\beta}$. The parameter α can be obtained by a linear fitting of the first experimental data points. Indeed, for small t, the analytical expression above can be approximated by

$$h(t) \cong \alpha t \sqrt{c_{A,i}c_{B,i}} / \rho$$

which shows that the slope of the experimental curve for t near zero is equal to $\alpha\sqrt{c_{A,i}c_{B,i}} / \rho$.
